# Development and evaluation of a blended educational programme for general practitioners’ trainers to stimulate proactive HIV testing

**DOI:** 10.1186/s12875-018-0723-8

**Published:** 2018-03-07

**Authors:** Ivo Kim Joore, Jan E. A. M. van Bergen, Gerben ter Riet, A. van der Maat, N. van Dijk

**Affiliations:** 1 Department of General Practice, Academic Medical Center, Amsterdam, The Netherlands; 2 Centre for Infectious Disease Control, National Institute for Public Health and the Environment (RIVM), Bilthoven, The Netherlands; 3 STI AIDS Netherlands (SOA AIDS Nederland), Amsterdam, The Netherlands

**Keywords:** Education, Medical continuing, General practitioners, Sexually transmitted diseases, Primary care, Prevention

## Abstract

**Background:**

In the Netherlands, a substantial proportion of newly diagnosed HIV patients present late for care, therefore, we investigated the effectiveness of a blended educational programme for trainers of GPs designed to stimulate proactive HIV testing.

**Methods:**

GP trainers at the Academic Medical Center in Amsterdam were invited to participate in a two days training programme incorporating evidence-based practice guidelines and multiple teaching strategies, including interactive lectures, discussion groups, e-learning and quality improvement targets. The GP trainers completed questionnaires before and after the programme to evaluate the effect of the programme. We also used six-monthly cumulative laboratory data from 2010 to 2015 to compare the participating GPs’ HIV tests to the general trend in testing among non-participating GPs.

**Results:**

150 GP trainers attended the first session, and 74 completed the questionnaires for both sessions. GPs median score on achieving their quality improvement targets was high and the quality of the programme highly appreciated. Between 2010 and 2013, the mean annual number of laboratory-documented HIV tests decreased by 9.1% in the 624 GPs in the control group, and by 13.0% for 11 GPs in the intervention group. After the programme, the annual decreases were 2.3% and 1.8%, respectively. Before the programme, the GPs in the intervention group had 50% more laboratory-documented HIV tests than GPs in the control group. After the programme, GPs in the intervention group had twice as many laboratory-documented HIV tests as the controls.

**Conclusions:**

We provided a detailed description of a programme based on educational and clinical evidence. We could not retrieve laboratory-documented HIV testing data for the majority of GPs in both the intervention and control groups. Therefore, the limited results should be interpreted with caution as our findings may not be representative of all participants. The blended educational programme appears to have stabilized – at a higher level – the initially stronger downward trend in testing for 11 GPs undergoing the intervention, indicating that the programme may have had an impact on their HIV testing behaviour.

**Electronic supplementary material:**

The online version of this article (10.1186/s12875-018-0723-8) contains supplementary material, which is available to authorized users.

## Background

It is estimated that, in the Netherlands, 12% of people with HIV have not been diagnosed [1, 2]. Moreover, 44% of newly diagnosed HIV patients present late for care, defined as a CD4+ T-cell count < 350/mm3, or presentation with an AIDS-defining illness, regardless of CD4 count [2]. Early preventive treatment for HIV has a public health benefit [3, 4], and initiating therapy after HIV diagnosis, regardless of CD4 count, has been found to improve the prognosis of the person being treated [5, 6], reinforcing the need for early detection.

Dutch general practitioners (GPs) act as gatekeepers to clinical care and are an important point of referral into specialized HIV care [7]. One-third of all HIV infections in the Netherlands are diagnosed by GPs [2, 8]. This underlines the important role of Dutch primary care in fighting the HIV epidemic.

The Dutch STI guidelines for GPs were released in 2013 [9, 10]. Educational strategies are often the first step in the implementation of evidence-based practice guidelines among GPs [11]. However, convincing GPs to follow new recommendations is a complex task [11–13]. Educational strategies are more effective in inducing behavioural change if they involve interactive training programmes that use multiple teaching strategies [12, 13]. A British study found higher HIV testing rates after a multifaceted educational programme [14]. There is also increasing interest in the use of internet-based learning in educational strategies [15, 16], while one previous study recommended blended learning, which combines electronic teaching methods with traditional teaching strategies [17]. A Dutch study showed that GPs appreciated a blended learning and found e-learning useful [18]. Therefore, we investigated whether a blended educational programme for GP trainers designed to stimulate proactive HIV testing would positively affect HIV testing in practice.

## Methods

We performed a prospective cohort study to evaluate a programme for GP trainers designed to stimulate proactive HIV testing.

### Context

From November 2013 to April 2014, 265 GP trainers at the Academic Medical Center, University of Amsterdam (AMC-UvA), were invited to participate in the programme. To become a GP, medical graduates must complete 3 years of specialty training linked with an experienced GP, the GP trainer. GP trainers complete a 4–5 year training programme, with 8 days of refresher training required every year. GP trainers are trained to perform a learning conversation with their resident during their 4-year training program as a trainer. Evidence-based practice guidelines for GP trainers are often referenced during this training [9]. These guidelines are developed by the Dutch College of GP trainers and are available on the internet to facilitate their use in daily practice [19].

### Content

The content of the programme was based on the Dutch STI guidelines and the accompanying e-learning package for GPs, which was released in September 2013 [9].

The STI guidelines promote more proactive HIV testing among men who have sex with men (MSM) and patients who originate from HIV endemic countries. A new provider-initiated HIV testing strategy is also briefly mentioned in the guidelines. This proactive strategy recommends offering an HIV test to individuals with HIV-related conditions, as supported by European guidelines [9, 20]. Another new provider-initiated HIV testing strategy is described in UK guidelines and is also mentioned in the additional file of the Dutch STI guidelines [10, 21–23]. This strategy recommends a ‘routine offer of HIV testing’ to all 15–59 year olds in primary care settings where HIV prevalence exceeds 2 in 1000. This strategy is operationalized either by offering an HIV test (a) when registering new patients or (b) to anyone who undergoes a blood test, regardless of the reason.

### Learning goals

These guidelines and their recommendations were used to formulate the goals and set-up of our blended learning programme. At the end of the programme, GP trainers were expected to have developed competences (knowledge, attitude, skills) that enabled them to diagnose HIV and other STIs (see Table 1).

**Table 1:** Learning objectives of the programme

Objective theme	Specific objective
Knowledge	GP trainers can explain the importance of early detection of HIV.
	GP trainers can explain the risk groups for HIV.
	GP trainers can explain STI related complaints.
	GP trainers have knowledge on how to perform a risk-assessment.
	GP trainers have an understanding of the different test locations of STI.
	GP trainers can explain the different questions asked in a sexual anamnesis.
	GP trainers can explain the role of the STI clinic in prevention and control of STI.
Attitude	GP trainers can express the important role of GP trainers in early detection of HIV.
	GP trainers can express the relevance of a risk-assessment.
	GP trainers acknowledge that an open attitude towards HIV and sexuality is important for prevention and control.
	GP trainers express a positive attitude towards proactive HIV testing.
Skills	GP trainers possesses skills to discuss sexual risk behavior and treatment in STI related consultations.
	GP trainers possesses skills to discuss proactive HIV testing.
	GP trainers are able to perform the right STI tests based on someone’s risk profile.
	GP trainers are able to explain how STI tests are executed.
	GP trainers possesses skills to refer to the STI clinic.

### Blended educational approach

Based on our learning goals for the programme and the experience of GP trainers, we also used evidence-based guidelines and multiple teaching strategies [9, 12, 13, 24, 25]. According to adult learning theory, active learner participation, multiple learning experiences and reflection on real-life experiences should be incorporated in educational strategies [25]. These were applied in our study design through e-learning, multiple meetings that focused on transferring knowledge, a ‘learning conversation’ with the GP’s own trainee, and focus groups to discuss barriers that were experienced in daily practice (see Table 2). The e-learning element was based on the STI guidelines for GPs and required about two hours. The online programme used various didactic methods, for example, interactive videos to change knowledge, attitude and behaviour concerning the STI consultation [9].

**Table 2:** Description of the blended educational programme

Blended educational programme			
	Duration	Content	Teaching methods
Preparation of the programme	90 min	GP trainers were asked to read the STI guideline and to do an e-learning before the start of the first meeting	
Training day 1	10 min	Introduction	Lecture
	5 min	Pre-test	Questionnaire (on knowledge and attitude)
	50 min	Information about the STI guideline and provider-initiated testing strategies	Interactive lecture with slides, videos and casuistry
	10 min	Summary & quality improvement targets, explanation of the homework assignments, evaluation and reflection of the first meeting	Lecture, questionnaire (quality improvement targets and on satisfaction)
Learning conversation with their resident			
Training day 2	15 min	A reflection on the preceding training day, what they had learned in daily practice (quality improvement targets) and on the learning conversation they had with their resident	lecture, discussion
	50 min	Discussion of barriers and facilitators related to new provider-initiated HIV testing	Focus groups
	10 min	Evaluation and reflection of the second meeting	Questionnaire
Post questionnaire	3 months after the end of the programme		Questionnaire

### Preparation of the programme

As independent learners, GP trainers were asked to read the STI guidelines and complete the e-learning package [9].

### The first meeting

The first meeting addressed the programme’s learning goals. Clinical knowledge and communication skills were also addressed in interactive workshops. GP trainers were randomly divided into small groups (12 to 25) designed to stimulate active learner participation and expose GP trainers to new and diverse real-life experiences. A member of the Department of General Practice at the AMC and an expert from the STI guidelines committee facilitated the groups.

### Learning conversation

As home-assignment after the first meeting, GP trainers were asked to formulate quality improvement targets which they executed the next six months in daily practice and to fill in the e-learning if they had not done this before. Three months after the first meeting a reminder of their home assignments was sent by e-mail. Also, GP trainers were asked to initiate a conversation with their own trainee, which would stimulate a further transference of knowledge, problem-solving skills and motivation for further learning [24, 25].

### Discussion groups

During the second meeting, GP trainers reflected on the first meeting and their personal real-life experiences in daily practice in focus groups and discussed the barriers and facilitators they encountered in applying the new guidelines and additional provider-initiated HIV testing strategies (published elsewhere [26]). This enhanced the opportunity to discuss and translate real-life issues into their own practice. These sessions were facilitated by staff and researchers from the Department of General Practice at the AMC who acted as moderators and observers. To ensure consistency in the way the programme was taught, all experienced and trained teachers (members of the Department of General Practice at the AMC and had no role in the design of the STI guideline) of both meetings were provided with a comprehensive handbook, including training materials.

### Laboratory-documented HIV testing

We evaluated the effect of the programme on participants’ frequency of laboratory-documented HIV testing over time. In the Netherlands, GPs collaborate with one or more laboratories, to which their patients are referred for HIV testing. We contacted every laboratory used by the participant GPs and requested information. After the initial contact, a second email was sent, including informed consent from the participating GPs, and we asked the laboratories to send us data on the total number of HIV tests for each GP over six-month periods between 2010 and 2015 (intervention group). We compared these figures with the average number of HIV tests among anonymized GPs performed in the laboratory every six months in the same period to determine whether the programme had an effect on the GP trainers’ use of laboratory-documented HIV testing (control group).

### Assessments and evaluation of the educational programme

To evaluate the effects of the programme on the GP trainers’ self-reported use of HIV testing and quality improvement targets, the participants were requested to complete a questionnaire focusing on the learning goals of the programme before and three months after the programme (Additional files 1, 2, 3 and 4). GP trainers were also asked to fill in a report form to evaluate both training meetings. To ensure content validity, the questionnaires were discussed with a senior epidemiologist who is specialised in medical education and GP training (NvD), a coordinator from the GP trainers educational program at the Department of General Practice at the Academic Medical Center of the University of Amsterdam (AvdM), and a GP specialised in STI/HIV in primary care (JvB). No further validation of the questionnaires has been performed. The questionnaire contained questions on:

1. Self-reported HIV testing items (*N* = 4). Questions on initiating an HIV test could be answered on 5-point Likert scale. Self-reported HIV testing in the previous three months was on an ordinal 6-point scale (6 = At least twice a week; 5 = once a week; 4 = 2–3 times a month; 3 = once monthly; 2 = 1–2 times in 3 months; 1 = never).
2. We performed a formative assessment by asking GP trainers to define their quality improvement targets after the first meeting. We asked them if they had achieved these goals at the second meeting, with a request to indicate on a 5-point Likert scale (1 = failure to 5 = success) whether they had implemented these goals in practice.
3. Knowledge items (*N* = 3). With binary (1) or multiple-choice answer options.
4. Attitude items (*N* = 7). The questions could be answered on a 5-point Likert scale.
5. Satisfaction with the meetings items (*N* = 12). These questions covered aspects such as the relevance of the content and the use of mixed teaching strategies. We used a 5-point Likert scale and open questions (“where 5 means strongly agree”).

### Statistical analysis

We assessed the six-monthly figures on HIV tests over time using a zero-inflated negative binomial regression model accounting for the large number of zeroes, over-dispersion and the clustering of tests within GPs using robust standard errors (zinb command [version 1.7.12] in STATA version 13.1 software). The effect of the educational programme was assessed using interaction terms between time (before/after programme) and programme (yes/no).

The scores for knowledge before the programme and after three months were compared using a McNemar test for categorical binary paired data. The knowledge-related questions were recoded from nominal categorical paired data into binary categorical paired data (correct/not correct answer). The scores for attitude-related questions, the initiation of HIV testing and self-reported HIV testing before and after three months were compared using a Wilcoxon signed rank test for categorical ordinal paired data. A *p*-value of < 0.05 was considered statistically significant. The median average score of all quality improvement targets was calculated. We used descriptive analysis for the evaluation of the two sessions (satisfaction). We analysed the questionnaire items using IBM SPSS version 22.0.

## Results

### Study population

In total, 265 GP trainers were invited to join the programme and 150 attended the first meeting, with 107 giving informed consent for participation in the scientific evaluation of the programme. Seventeen GP trainers were excluded because they only participated in the first meeting. The demographic characteristics of the remaining 90 GP trainers are described in Table 3. Of these, 74/90 (82.2%) completed both questionnaires and 50% (74/150) off all GPs who attended the first meeting participated in the study. Before the start of the programme, 9.5% (7/74) of the GP trainers had completed the e-learning package, compared to 67.6% (50/74) three months after the second meeting. In addition, 79.7% (59/74) reported having had a learning conversation with their trainee after the second meeting.

**Table 3:** Demographic Characteristics of the total group of GP trainers

	N	%
Total	90	
Male	45	50.0
Age, (IOR)	51 (46;57)	
Location of practice		
City	70	77.7
Country side	20	22.2
Years of experience as GP		
< 10 years	16	17.8
11–15 years	25	27.8
16–20 years	15	16.7
20–25 years	14	15.6
26–30 years	11	12.2
> 30 years	9	10.0
Years of experience as GP trainer (quartiles)	6	[3, 9]
Number of HIV patients in practice		
< 5 patients	55	62.5
5–10 patients	22	25.0
10–25 patients	5	5.7
> 25 patients	6	6.8
Missing	1	
Number of patients diagnosed with HIV in the past year (quartiles)	1	[0,1]

### GP trainers’ laboratory-documented HIV testing

Of the 90 GP trainers, 78 listed one or more laboratories (1–3) where they sent patients for HIV testing. In total, they mentioned 21 laboratories. These laboratories were approached multiple times. While we received information from seven laboratories, only four sent complete information. GPs in the intervention or control groups were only included if we had complete information from the laboratories. As a result, 11 GP trainers participating in the educational programme were included for further analysis. Some laboratories refused to participate because they had not been involved at the start of the study, or did not responded because the research was of low priority for them, or because they had difficulties retrieving this information from their database.

**Fig. 1:**
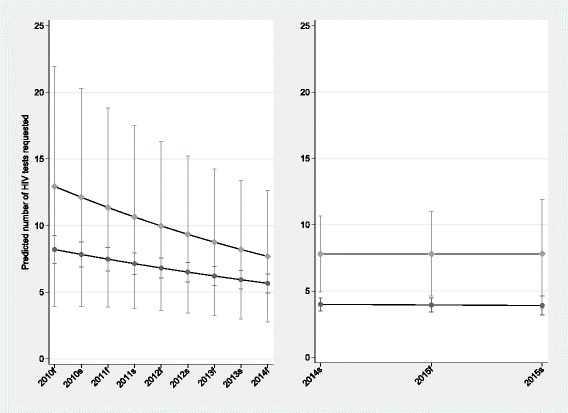
Mean number of HIV tests requested by 635 Dutch General Practitioners before (2010–2013) and after (2014–2015) a blended STI educational programme in the first half of 2014

We found that, between 2010 and 2013, the mean annual number of laboratory-documented HIV tests decreased by 9.1% for the 624 control GPs, and by 13.0% for the 11 intervention GPs. After the programme, the annual decreases were 2.3% and 1.8% for the control and intervention groups, respectively. Before the programme, GPs in the intervention group had 50% more laboratory-documented HIV tests than the controls (IRR 1.50; 95% CI 0.79–2.85; *p* = 0.211). After the programme, the intervention group had twice as many laboratory-documented HIV tests as the controls (IRR 2.05; 95% CI 1.35–3.12; *p* = 0.001) (p_interaction-pre- programme_ = 0.198; p_interaction-post- programme_ = 0.041), (see Fig. 1).

### Self-reported HIV testing

Self-reported HIV testing behaviour showed a significant decrease after the training programme (*p* = 0.004). Before the programme, the HIV test was significantly more often initiated by the patient than after the programme (*p* = 0.001) (see Table 4). There was an increase in the frequency HIV tests initiated on the basis of symptoms or complaints related to an HIV-related infection (*p* = 0.017).

**Table 4:** Self-reported HIV testing and initiatives of the HIV test in the past three months

	Mean (sd) score at the start of the programme (n = 74)	Mean (sd) score after three months of the programme (*n* = 74)	*p*-value
Self-reported HIV testing behaviour 6-point scale (scale 1–6: never - at least twice a week).	3.3 (1.3)	2.9 (1.1)	0.004
HIV test initiated by the patient (scale 1–5; not-always)	3.8 (0.9)	3.3 (1.0)	0.001
HIV test initiated by GP trainers (scale 1–5; not-always)	2.1 (0.9)	2.2 (0.7)	0.568
HIV test initiated by symptoms or complaints (scale 1–5; not-always)	1.5 (0.6)	1.7 (0.7)	0.017

### Intended behavioural change

Of 74 GP trainers, 69 defined one or more (1–3) quality improvement targets. In addition, 179 change in behaviour goals were defined and the overall median average score on achieving their goals was 4 (1 = failure to 5 = success). Most of these goals were related to focusing more on sexual history and behaviour, STI tests and the location of testing and HIV-related conditions (see Table 5).

**Table 5:** Categories of quality improvement targets

Categories	Number of quality improvement targets per category
Sexual history and techniques	30
Ask more in-depth questions	6
Talk more about sex	6
Be aware of symptoms and conditions related to HIV/STI	17
Be aware of new HIV/STI tests	22
Different body-locations to test the patient	16
More proactive HIV testing	15
Awareness of HIV testing among patients from HIV endemic countries	5
Awareness of HIV testing among MSM	6
Give instruction to resident	14
More often test the BIG five	8
Awareness of window phase	3
Make the e-learning of the STI guideline	7
Awareness of not having sex after treatment	3
Patient information about STI/HIV	2
Applying of specific obtained knowledge	10
Curation, Counselling and Contact sex partners	7
Take into account own risk payments	1
Prevention of STI/HIV among adolescents	1

### Scores on knowledge and attitude-related questions

The proportion of GP trainers that answered the knowledge-related questions correctly was significantly higher after the programme for every question (see Table 6). Also, we compared the mean attitude scores for the related questions, but minimal to no significant differences were observed.

**Table 6:** Percentage on the questions about knowledge and mean Likert-scores on the questions about attitude

Knowledge	Pre-training % of GP trainers with correct answer	Post-training % of GP trainers with correct answer	*p*-value
• ‘Is HIV testing required in high-risk groups?’	83.6	95.9	0.012
• ‘What is the percentage of people unaware of their HIV infection?’	41.1	78.1	0.000
• ‘What is the percentage of people diagnosed late for care?’	42.9	72.9	0.000
Attitude			
	Mean (sd) score at the start of the programme (n = 74)	Mean (sd) score after three months of the programme (n = 74)	*p*-value
• Proactive HIV testing is a task of the GP	3.9 (0.8)	3.9 (0.6)	0.744
• It is a task of the GP to be aware of the patients sexual orientation	3.5 (0.9)	3.5 (0.8)	0.566
• Sexual history and techniques is an essential part of a STI consultation	4.1 (0.6)	4.3 (0.5)	0.105
• Lack of time to discuss a sexual history and techniques in a STI consultation	2.7 (0.9)	2.9 (0.9)	0.197
• I rather refer patients with STI symptoms to a STI clinic	2.2 (0.9)	2.0 (0.7)	0.038
• It’s unacceptable to discuss an HIV test if patients visit their GP with no STI related questions	2.4 (0.8)	2.4 (0.7)	0.902
• It’s a GP trainers task to register sexual orientation	2.9 (1.0)	2.7 (0.9)	0.187

### Satisfaction of the GP trainers with both meetings

The total satisfaction value of both meetings within the programme was rated high, at an overall median average score of 4 (scale 1 to 5 “where 5 means strongly agree”) on both days. The relevance of each day in relation to the content of the programme and the use of mixed teaching strategies had a similar score. GP trainers were satisfied and enthusiastic about their teachers’ performance on both days, with an average of 4 to 5 (scale 1 to 5 “where 5 means strongly agree”). GP trainers mentioned that the day was interactive, informative and focused on clinical practice (as reported in the open questions section).

## Discussion

This study describes the development and evaluation of an evidence-based blended educational programme for GP trainers designed to stimulate proactive HIV testing. The median score of GP trainers on achieving their quality improvement targets was high during the programme. The meetings were also highly appreciated. Our findings present weak evidence of an effect of the programme on GPs’ laboratory-documented HIV testing. Before the programme, the GPs had 50% more laboratory-documented HIV tests than those in the control group. After the programme, the GPs had twice as many laboratory-documented HIV tests as the controls. Despite recommendations in the 2013 guidelines on proactive HIV testing, we observed a continuing downward trend in GPs’ laboratory-documented HIV testing.

### Comparison with existing literature

There is no gold standard for successful implementation of new recommendations in evidence-based guidelines [11]. It is recommended that educational strategies should be interactive and include multiple teaching strategies, for example, group discussions about evidence and setting quality improvement targets [12]. We integrated multiple teaching strategies but found only a moderate effect on GP trainers’ laboratory-documented HIV testing behaviour. This concurs with other evidence on the difficulties of changing physicians’ behaviour [11–13]. GP trainers in our programme mentioned various barriers to and facilitators of new proactive HIV testing strategies recommended by guidelines [26]. Integrating knowledge of these barriers into the design of future interventions may help in the implementation of new proactive HIV testing strategies in daily practice [12]. Understanding the barriers hindering the adoption of new recommendations of guidelines is important and strategies should be tailored to addressing them.

Part of our study design involved active learner participation, for example, GP trainers had to formulate their own quality improvement targets [27, 28]. These goals were designed to encourage the transfer of new knowledge to daily practice by using quality improvement targets for behaviour change. We showed that a high overall median score for achieving rate of implementing GP trainers formulated intended changes.

The theory of reasoned action states that attitude and subjective norms are related (predictors) to behaviour [29]. We found no change in the majority of the attitude items. One explanation could be that the attitude items in our study were not representative of the observed behavioural change. Interestingly, the attitude item, ‘I would rather refer patients with STI symptoms to an STI clinic’, significantly decreased after the programme, which may indicate that GP trainers were more familiar with this topic.

A Dutch study showed that a multifaceted training programme using guidelines, feedback and social interaction resulted in modest improvements in test ordering by GPs [30]. Feedback on the performance of laboratory-test ordering was considered as a follow-up to our study design but was unsuccessful due to difficulties in retrieving GP trainers’ laboratory-documented HIV testing results. New efforts are being made in the Netherlands to collect this useful information. In 2014, the ‘HIV Transmission Elimination AMsterdam’ programme (H-TEAM) was set up [31]. The H-TEAM develops and implements a mix of interventions to prevent the spread of the virus and to extend HIV testing and direct linkage to care. For example, an educational programme for GPs in Amsterdam has been developed that integrates feedback on HIV test performance to stimulate proactive HIV testing.

We reported that before the programme, the GP trainers intervention group had 50% more laboratory-documented HIV tests than the controls. This might be explained by the fact that GP trainers who participated in the study had more interest in HIV prevention and were thus more likely to test patients for HIV compared to the control group.

The educational programme did have an impact on the testing behaviours of the participating GPs, but the overall downward trend in HIV tests among GPs was surprising. Even before the 2013 guidelines were established, the professional media was drawing attention to a need to increase proactive HIV testing [3, 4]. There are several reasons that might explain this counterintuitive downward trend. People from risk groups could have changed their healthcare seeking behaviour and decided to be tested for HIV at an STI clinic rather than at the GP. Another explanation is that due to the compulsory excess payments in primary care, heterosexual people are more reluctant to undertake HIV testing than before [32, 33]. Also, we hypothesize that GPs target the right group who should be tested. More research is warranted to find out the reason for this downward HIV testing trend.

### Strengths and limitations

One strength of this study is that we initiated a blended education programme that incorporated evidence-based practice guidelines and multiple evidence-based teaching strategies to stimulate GP trainers’ HIV testing behaviour.

Also, we tried to incorporate GPs’ laboratory-documented HIV testing to objectively measure any change in behaviour. Laboratory-documented HIV testing provides more detailed information about GPs’ actual testing behaviour compared to information gained from questionnaires, as self-reported levels of HIV testing may be biased by socially desirable answers or recall bias.

We also collected information on laboratory-documented HIV testing trends for a prolonged period and not only before and after the programme. This enabled us to reflect in greater depth on the outcomes, rather than only relying on an analysis of the questionnaires. Self-reported HIV testing in the questionnaires suggested a decrease in HIV testing. However, the programme appeared to have stabilized – at a higher level of request for laboratory-documented HIV tests – the initially stronger downward trend in the 11 GPs who participated in the programme.

One limitation of the study is that we could not collect data on laboratory-documented HIV testing for the majority of GPs in both the intervention and control groups due to lack of cooperation from the laboratories. Therefore, answering the main research question if the educational programme was effective was severely hampered. The limited results should be interpreted with caution as our findings may not be representative of all participants. It was not possible to distinguish a decline in the number of HIV tests per patient from a decline in the number of patients treated by the GP. Also, we had no insight in the stability of the population of GPs who use the four laboratories. A decrease in the number of GPs would reduce the number of patients and subsequently effect the number of HIV tests. Also, we acknowledge that GPs could have gradually switching their patients’ HIV tests to the 17 other laboratories. More detailed information needs to be collected from the laboratories, as described above, for evaluation of educational programmes. We vigorously attempted to retrieve this laboratory information but even after multiple reminders by phone and email not all laboratories agreed or responded to our requests. This is a serious drawback faced when implementing a study-design in daily reality. We recommend that laboratories need to be more involved in the design of the study. This could help bypassing some of the barriers regarding the collection of the data. Another way to evaluate these programmes is to use general practice-documented HIV testing data. As a different level of use of laboratory-documented HIV testing by GPs after the programme only offers partial evidence of GPs following the new recommendations. We would also like to know which patients are tested (for example, MSM versus non-MSM) and why (for example, provider initiated testing versus patient-initiated testing). Future research needs to collect this additional information to determine the actual impact of an educational programme on GPs’ HIV testing behaviour.

## Conclusions

We provided a detailed description of a programme based on educational and clinical evidence. We could not retrieve laboratory-documented HIV testing data for the majority of GPs in both the intervention and control groups. Therefore, the limited results should be interpreted with caution as our findings may not be representative of all participants. The blended educational programme appears to have stabilized – at a higher level – the initially stronger downward trend in testing for 11 GPs undergoing the intervention, indicating that the programme may have had an impact on their HIV testing behaviour.

## Additional files

Additional file 1: Pre-test questionnaire. (DOC 64 kb)

Additional file 2: Report form Quality improvement targets. (DOC 24 kb)

Additional file 3: Post Questionnaire. (DOC 50 kb)

Additional file 4: Evaluation forms training day 1 and 2. (DOC 31 kb)

## Abbreviations

AIDS: Acquired immunodeficiency syndrome
AMC-UvA: Academic Medical Center, University of Amsterdam
CI: Confidence interval
GP: General practitioner
HIV: Human immunodeficiency virus
H-TEAM: HIV Transmission Elimination Amsterdam
IRR: Incidence rate ratio
MSM: Men who have sex with men
STI: Sexually transmitted infection
UK: United Kingdom
