# Supplementary material for: Development and evaluation of a blended educational programme for general practitioners’ trainers to stimulate proactive HIV testing
Source: BMC Fam Pract. 2018 Mar 7;19:36. doi: 10.1186/s12875-018-0723-8 (PMC5842561; doi:10.1186/s12875-018-0723-8)
Supplement: Supplementary file 1 — Pre-test questionnaire. (DOC 64 kb) [file 12875_2018_723_MOESM1_ESM.doc]

**Pre-test questionnaire**

GP name:

Research number:

The location of practice (city or countryside)*:

GPs name will be removed and only anonymised data can be used by the researcher.

1. Did you participate in the e-learning of the Dutch STI guideline? Circle the answer that applies.

- Yes
- No

1. What is your gender? Circle the answer that applies.

- Male
- Female

1. What is your of birth?

……..

1. How many years of work experience do you have as a GP? Circle the answer that applies.
   - Less than 10 years
   - 11-15 years
   - 16-20 years
   - 20-25 years
   - years
   - More than 30 years
2. How many years of experience do you have as a GP trainer?

…..Years

1. How many HIV patients do you have in your practice? Circle the answer that applies.

- Less than 5 patients
- 5-10 patients
- 10-25 patients
- More than 25 patients
- Missing

1. How many patients were diagnosed with HIV in the past year in your practice?

….Patients

1. What is your self-reported HIV/STI testing behaviour in the past three months? Cross the correct answer(s).

|  | Never | 1-2 times in 3 months | Once monthly | 2-3 times a month | Once a week | At least twice a week |
| --- | --- | --- | --- | --- | --- | --- |
| Chlamydia |  |  |  |  |  |  |
| Gonorrhea |  |  |  |  |  |  |
| Syphilis |  |  |  |  |  |  |
| HIV |  |  |  |  |  |  |
| Hepatitis B |  |  |  |  |  |  |
| Genital Herpes |  |  |  |  |  |  |
| Trichomonas |  |  |  |  |  |  |

1. What was the reason for the HIV test? Circle the answer that applies.
2. Initiated by the patient

- Not
- Sometimes
- Regularly
- Often
- Always

1. Initiated by GP

- Not
- Sometimes
- Regularly
- Often
- Always

1. Initiated by symptoms or complaints

- Not
- Sometimes
- Regularly
- Often
- Always

1. Which tests would you do in your practice among high risk patients and low risk patients? Cross the correct answer(s).

|  | High risk patients | Low risk patients |
| --- | --- | --- |
| HIV |  |  |
| Syphilis |  |  |
| Gonorrhea |  |  |
| Chlamydia |  |  |
| Hepatitis B |  |  |

1. What is the percentage of people unaware of their HIV infection? Circle the answer that applies.

- <5%
- 5-15%
- 30-40%
- >50%

1. What is the percentage of people diagnosed late for care? Circle the answer that applies.

- < 5%
- 5-15%
- 40-50%
- >70%

14. In the following statements, indicate the extent to which you agree with the statement. Circle the answer that applies.

- Proactive HIV testing is a task of the GP

| ○ Strongly disagree | ○ Disagree | ○ Neither agree nor disagree | ○ Agree | ○ Strongly agree |
| --- | --- | --- | --- | --- |

- It is a task of the GP to be aware of the patients sexual orientation

| ○ Strongly disagree | ○ Disagree | ○ Neither agree nor disagree | ○ Agree | ○ Strongly agree |
| --- | --- | --- | --- | --- |

- Sexual history and techniques is an essential part of a STI consultation.

| ○ Strongly disagree | ○ Disagree | ○ Neither agree nor disagree | ○ Agree | ○ Strongly agree |
| --- | --- | --- | --- | --- |

- Lack of time to discuss a sexual history and techniques in a consultation

| ○ Strongly disagree | ○ Disagree | ○ Neither agree nor disagree | ○ Agree | ○ Strongly agree |
| --- | --- | --- | --- | --- |

- I rather refer patients with STI symptoms to a STI clinic

| ○ Strongly disagree | ○ Disagree | ○ Neither agree nor disagree | ○ Agree | ○ Strongly agree |
| --- | --- | --- | --- | --- |

- It’s unacceptable to discuss an HIV test if patients visit their GP with no STI related questions

| ○ Strongly disagree | ○ Disagree | ○ Neither agree nor disagree | ○ Agree | ○ Strongly agree |
| --- | --- | --- | --- | --- |

- It’s a GP trainers task to register sexual orientation.

| ○ Strongly disagree | ○ Disagree | ○ Neither agree nor disagree | ○ Agree | ○ Strongly agree |
| --- | --- | --- | --- | --- |

*The location of practice was determined if a practice was located in or outside a city.

Thank you very much for your cooperation
