# Supplementary material for: Development and evaluation of a blended educational programme for general practitioners’ trainers to stimulate proactive HIV testing
Source: BMC Fam Pract. 2018 Mar 7;19:36. doi: 10.1186/s12875-018-0723-8 (PMC5842561; doi:10.1186/s12875-018-0723-8)
Supplement: Supplementary file 2 — Report form Quality improvement targets. (DOC 24 kb) [file 12875_2018_723_MOESM2_ESM.doc]

**Quality improvement targets**

Training day 1

GP name:

Research number:

GPs name will be removed and only anonymised data can be used by the researcher.

Following the topic on the new STI guideline, I have decided to make the following quality improvement targets:

1…………………………………….

2.……………………………………

3.…………………………………….

**Quality improvement targets**

Training day 2

GP name:

Research number:

GPs name will be removed and only anonymised data can be used by the researcher.

The quality improvement targets you formulated on training day 1 are shown down below. Please answer the questions.

Following the topic on the new STI guideline, I have decided to make the following quality improvement targets:

1…………………………………….

Have you succeeded in implementing this quality improvement target? Cross the correct answer(s).

No 1 2 3 4 5 Yes

❑ ❑ ❑ ❑ ❑

2.……………………………………

Have you succeeded in implementing this quality improvement target? Cross the correct answer(s).

No 1 2 3 4 5 Yes

❑ ❑ ❑ ❑ ❑

3.…………………………………….

Have you succeeded in implementing this quality improvement target? Cross the correct answer(s).

No 1 2 3 4 5 Yes

❑ ❑ ❑ ❑ ❑
