# Supplementary material for: Development and evaluation of a blended educational programme for general practitioners’ trainers to stimulate proactive HIV testing
Source: BMC Fam Pract. 2018 Mar 7;19:36. doi: 10.1186/s12875-018-0723-8 (PMC5842561; doi:10.1186/s12875-018-0723-8)
Supplement: Supplementary file 3 — Post Questionnaire. (DOC 50 kb) [file 12875_2018_723_MOESM3_ESM.doc]

**Post questionnaire**

GP name:

Research number:

GPs name will be removed and only anonymised data can be used by the researcher.

1. Did you participate in the e-learning of the Dutch STI guideline? Circle the answer that applies.

- Yes
- No

1. Did you have a learning conversation on STI/HIV with your trainee after the second meeting? Circle the answer that applies.

- Yes
- No

1. What is your self-reported HIV/STI testing behaviour in the past three months? Cross the correct answer(s).

|  | Never | 1-2 times in 3 months | Once monthly | 2-3 times a month | Once a week | At least twice a week |
| --- | --- | --- | --- | --- | --- | --- |
| Chlamydia |  |  |  |  |  |  |
| Gonorrhea |  |  |  |  |  |  |
| Syphilis |  |  |  |  |  |  |
| HIV |  |  |  |  |  |  |
| Hepatitis B |  |  |  |  |  |  |
| Genital Herpes |  |  |  |  |  |  |
| Trichomonas |  |  |  |  |  |  |

1. What was the reason for the HIV test? Circle the answer that applies.
2. Initiated by the patient

Not

Sometimes

Regularly

Often

Always

1. Initiated by GP

Not

Sometimes

Regularly

Often

Always

1. Initiated by symptoms or complaints

Not

Sometimes

Regularly

Often

Always

1. Which tests would you do in your practice among high risk patients and low risk patients? Cross the correct answer(s).

|  | High risk patients | Low risk patients |
| --- | --- | --- |
| HIV |  |  |
| Syphilis |  |  |
| Gonorrhea |  |  |
| Chlamydia |  |  |
| Hepatitis B |  |  |

1. What is the percentage of people unaware of their HIV infection? Circle the answer that applies.

<5%

5-15%

30-40%

>50%

1. What is the percentage of people diagnosed late for care? Circle the answer that applies.

< 5%

5-15%

40-50%

>70%

1. In the following statements, indicate the extent to which you agree with the statement. Circle the answer that applies.

- Proactive HIV testing is a task of the GP

| ○ Strongly disagree | ○ Disagree | ○ Neither agree nor disagree | ○ Agree | ○ Strongly agree |
| --- | --- | --- | --- | --- |

- It is a task of the GP to be aware of the patients sexual orientation

| ○ Strongly disagree | ○ Disagree | ○ Neither agree nor disagree | ○ Agree | ○ Strongly agree |
| --- | --- | --- | --- | --- |

- Sexual history and techniques is an essential part of a STI consultation.

| ○ Strongly disagree | ○ Disagree | ○ Neither agree nor disagree | ○ Agree | ○ Strongly agree |
| --- | --- | --- | --- | --- |

- Lack of time to discuss a sexual history and techniques in a consultation

| ○ Strongly disagree | ○ Disagree | ○ Neither agree nor disagree | ○ Agree | ○ Strongly agree |
| --- | --- | --- | --- | --- |

- I rather refer patients with STI symptoms to a STI clinic

| ○ Strongly disagree | ○ Disagree | ○ Neither agree nor disagree | ○ Agree | ○ Strongly agree |
| --- | --- | --- | --- | --- |

- It’s unacceptable to discuss an HIV test if patients visit their GP with no STI related questions

| ○ Strongly disagree | ○ Disagree | ○ Neither agree nor disagree | ○ Agree | ○ Strongly agree |
| --- | --- | --- | --- | --- |

- It’s a GP trainers task to register sexual orientation.

| ○ Strongly disagree | ○ Disagree | ○ Neither agree nor disagree | ○ Agree | ○ Strongly agree |
| --- | --- | --- | --- | --- |

Thank you very much for your cooperation
