# Supplementary material for: Development and evaluation of a blended educational programme for general practitioners’ trainers to stimulate proactive HIV testing
Source: BMC Fam Pract. 2018 Mar 7;19:36. doi: 10.1186/s12875-018-0723-8 (PMC5842561; doi:10.1186/s12875-018-0723-8)
Supplement: Supplementary file 4 — Evaluation forms training day 1 and 2. (DOC 31 kb) [file 12875_2018_723_MOESM4_ESM.doc]

**Evaluation form**

GP name:

Research number:

Date:

GPs name will be removed and only anonymised data can be used by the researcher.

How do you rate the following aspects? Fill in the correct answer.

1 = strongly disagree or failure

5= strongly agree or success

| Teacher  Enthusiastic inspiring input  Degree of interaction of the teacher with the group  Quality of contribution  Translating their own expertise to their general practice  Meeting  Topic matches the learning needs  Relevance of the day in relation to the content of the programme  Content of the topic  Work form (use of mixed teaching strategies)  How do you look back on the total satisfaction of the meeting |  | 1 2 3 4 5  ❑ ❑ ❑ ❑ ❑  ❑ ❑ ❑ ❑ ❑  ❑ ❑ ❑ ❑ ❑  ❑ ❑ ❑ ❑ ❑    1 2 3 4 5  ❑ ❑ ❑ ❑ ❑  ❑ ❑ ❑ ❑ ❑  ❑ ❑ ❑ ❑ ❑  ❑ ❑ ❑ ❑ ❑  ❑ ❑ ❑ ❑ ❑ |
| --- | --- | --- |
|  |  |  |

What were the strong points of the meeting?

|  |
| --- |

What should change according to you?

|  |
| --- |

Feedback for the teacher

What went well and what could be better in your opinion?

|  |
| --- |
